# ﻿Morphology and molecular analyses reveal three new species of Botryosphaeriales isolated from diseased plant branches in China

**DOI:** 10.3897/mycokeys.97.102653

**Published:** 2023-04-26

**Authors:** Lu Lin, Yukun Bai, Meng Pan, Chengming Tian, Xinlei Fan

**Affiliations:** 1 The Key Laboratory for Silviculture and Conservation of Ministry of Education, Beijing Forestry University, Beijing 100083, China Beijing Forestry University Beijing China

**Keywords:** *Aplosporella*, dieback, *Dothiorella*, *Phaeobotryon*, phylogeny, taxonomy

## Abstract

The Botryosphaeriales represents an ecologically diverse group of fungi, comprising endophytes, saprobes, and plant pathogens. In this study, taxonomic analyses were conducted based on morphological characteristics and phylogenetic analyses of multi-gene sequence data from four loci (ITS, LSU, *tef1*-α, and *tub2*). Thirteen isolates obtained from Beijing and Yunnan Province were identified as seven species of Botryosphaeriales, including *Aplosporellajaveedii*, *Dothiorellaalpina*, *Phaeobotryonaplosporum* and *Ph.rhois*, and three previously undescribed species, namely *Aplosporellayanqingensis*, *Dothiorellabaihuashanensis*, and *Phaeobotryonplatycladi*. Additionally, the new records of *Dothiorellaalpina* from the host species *Populusszechuanica*, *Phaeobotryonaplosporum* from *Juglansmandshurica*, and *Phaeobotryonrhois* from Populusalbavar.pyramidalis are included.

## ﻿Introduction

The Botryosphaeriales C.L. Schoch, Crous & Shoemaker is an ecologically diverse fungal order comprising endophytes, saprobes, and plant pathogens (Schoch et al. 2006; Ekanayaka et al. 2016; Phillips et al. 2019). Slippers et al. (2013) provided molecular and morphological evidence to show that the Botryosphaeriales included six families (Aplosporellaceae Slippers, Boissin & Crous, Botryosphaeriaceae Theiss. & Syd., Melanopsaceae A.J.L. Phillips, Slippers, Boissin & Crous, Phyllostictaceae Fr., Planistromellaceae M.E. Barr, and Saccharataceae Slippers, Boissin & Crous). Then, Wyka and Broders (2016) introduced Septorioideaceae Wyka & Broders., Yang et al. (2017) introduced two new families (Endomelanconiopsisaceae Tao Yang & Crous and Pseudofusicoccumaceae Tao Yang & Crous). However, Phillips et al. (2019) argued that only six families (Aplosporellaceae, Botryosphaeriaceae, Melanopsaceae, Phyllostictaceae, Planistromellaceae and Saccharataceae) could be accepted in Botryosphaeriales, with reducing Endomelanconiopsisaceae, Pseudofusicoccumaceae, and Septorioideaceae to the synonymy under Botryosphaeriaceae, Phyllostictaceae, and Saccharataceae, respectively. In the present study, thirteen isolates were classified as three genera (*Aplosporella* Speg., *Botryosphaeria* Ces. & De Not., and *Phaeobotryon* Theiss. & Syd.) in two families (Aplosporellaceae and Botryosphaeriaceae).

Aplosporellaceae was introduced by Slippers et al. (2013) to accommodate two genera viz. *Aplosporella* and *Bagnisiella* Speg. However, Slippers et al. (2013) suggested that *Aplosporella* and *Bagnisiella* should be synonymized based on their close phylogenetic relationships and their remarkably similar multiloculate sporocarps. Ekanayaka et al. (2016) agreed with this and provided evidence that the sexual morph of *Aplosporellathailandica* Ekanayaka, Dissanayaka, Q. Zhao & K.D. Hyde resembles *Bagnisiella*. Phillips et al. (2019) formally placed *Bagnisiella* as a synonym of *Aplosporella*. Sharma et al. (2017) introduced *Alanomyces* Roh. Sharma in Aplosporellaceae based on four loci phylogeny. Therefore, two genera (*Alanomyces* and *Aplosporella*) can be accepted in Aplosporellaceae. The morphological characters of *Aplosporellaceae* are multiloculate ascostromata, septate pseudoparaphyses, aseptate and ellipsoid to ovoid ascospores, and ellipsoid to subcylindrical and hyaline to pigmented conidia (Slippers et al. 2013; Phillips et al. 2019).

Botryosphaeriaceae was introduced by Theissen and Sydow (1918) for three genera (*Botryosphaeria*, *Phaeobotryon*, and *Dibotryon* Theiss. & Syd.). Over the years the family and genera have undergone several taxonomic revisions and updates. Currently, the Botryosphaeriaceae has approximately 100 verified species in 24 genera, according to DNA sequence data (Phillips et al. 2013; Slippers et al. 2013; Yang et al. 2017; Xiao et al. 2021; Zhang et al. 2021). *Botryosphaeria* has uniloculate and clustered ascostromata and septate pseudoparaphyses (Phillips et al. 2019). In the phylogenetic tree of Botryosphaeriaceae, hyaline or colored conidia or ascospores are distributed randomly (Slippers et al. 2013). A large number of new species have been described in recent years, which indicated that the diversity of Botryosphaeriaceae was worthy of further exploration (Bezerra et al. 2021; Zhang et al. 2021; Sun et al. 2022).

With the modern taxonomic approaches applying, more than 30 novel species have been identified in the last five years (Zhang et al. 2021; Rathnayaka et al. 2022; Sun et al. 2022; Wang et al. 2023). Considering the important economic status of Botryosphaeriales, a survey to explore more hidden species of Botryosphaeriales was considered imperative. Thus, a survey on the diversity of Botryosphaeriales on diseased branches was conducted in Beijing and Yunnan Province from 2021 to 2022. In this study, we introduce three new species, in which *Aplosporellayanqingensis* and *Phaeobotryonplatycladi* were collected from *Platycladusorientalis* and *Dothiorellabaihuashanensis* were collected from *Juniperuschinensis* in China. Moreover, the newly discovered *Dothiorellaalpina* from *Populusszechuanica*, *Phaeobotryonaplosporum* from *Juglansmandshurica*, and *Ph.rhois* from Populusalbavar.pyramidalis are featured.

## ﻿Materials and methods

### ﻿Fungal isolation

Fresh specimens (woody branches and twigs with canker or dieback symptoms) were randomly collected in Beijing and Yunnan Province from the summer of 2021 to the autumn of 2022. The specimens were packed in kraft paper bags and transferred to the laboratory for fungal isolation following Jiang et al. (2022). Isolates were obtained by removing the spore mass from conidiomata to sterilised distilled water using sterilised needle, and generating single spore colonies on potato dextrose agar (PDA: 200 g potatoes, 20 g dextrose, 20 g agar per L) at 25 °C in the dark. After three to five days, hyphal tips were transferred to new PDA plates twice to obtain a pure culture. The cultures are deposited in the China Forestry Culture Collection Center (**CFCC**; http://www.cfcc-caf.org.cn/), and the specimens in this study are deposited in the Museum of the Beijing Forestry University (**BJFC**).

### ﻿Morphology

Morphological observations were conducted based on conidiomata produced on infected plant tissues. The conidiomata were manually sectioned using a double-edged blade and examined under a dissecting microscope for macroscopic and microscopic characterization, while conidiomata structure and size were imaged with a Leica stereomicroscope (M205) (Leica Microsystems, Wetzlar, Germany). Conidia and other microstructures were selected randomly for observation using a Nikon Eclipse 80i microscope (Nikon Corporation, Tokyo, Japan) equipped with a Nikon digital sight DSRi2 high-definition colour camera with differential interference contrast (DIC). Fifty conidia were measured per species, and 30 measurements were taken of other morphological structures. Colony characters i.e. colours and texture on PDA and MEA (malt extract agar; 30 g malt extract, 5 g mycological peptone, 15 g agar per L) at 25 °C were observed and noted over 14 days. The colony colours were determined based on the colour charts of Rayner (1970).

### ﻿DNA extraction, amplification and sequencing

The fresh mycelium from PDA was scraped and put it in a 1.5 mL centrifuge tube for genomic DNA extraction which used the modified CTAB (cetyltrimethylammonium bromide) method (Doyle and Doyle 1990). For initial species confirmation, the internal transcribed spacer (ITS) region was sequenced using the primer pairs ITS1/ITS4 (White et al. 1990) for all isolates. The BLAST tool (https://blast.ncbi.nlm.nih.gov/Blast.cgi) was used to compare the resulting sequences with those in GenBank. After confirmation to the genus level, additional partial loci were amplified, including the nuclear ribosomal large subunit (LSU), the partial translation elongation factor 1-alpha (*tef1*-α), and partial beta-tubulin (*tub2*) using the primer pairs LR0R/LR5 (Vilgalys and Hester 1990), EF1-728F/EF1-986R (Carbone and Kohn 1999), and Bt2a/Bt2b (Glass and Donaldson 1995), respectively. The additional combination of T1 and Bt2b (Glass and Donaldson 1995; O’Donnell and Cigelnik 1997) was used in case of amplification failure of the primer Bt2a and Bt2b. The genes used in different genera and the amplification conditions are listed in Table 1. The PCR mixture for all regions consisted of 1 µL DNA template, 1 µL each 10 µM primer, 10 µL T5 Super PCR Mix (containing Taq polymerase, dNTP and Mg^2+^, Beijing TisingKe Biotech Co., Ltd., Beijing, China), and 7 µL sterile water. PCR products were electrophoresed in 1% agarose gel and the DNA was sequenced by the SinoGenoMax Company Limited (Beijing, China). The forward and reverse reads were edited and assembled with Seqman v. 7.1.0 in the DNASTAR Lasergene core suite software (DNASTAR Inc., Madison, Wisconsin USA). All sequences generated in this study were submitted to GenBank (Suppl. material 1).

**Table 1.** Genes used in this study with PCR primers and optimal annealing temperature.

Locus	PCR primers	PCR: thermal cycles: (Annealing temp. in bold)	Genus
ITS	ITS1/ITS4	(95 °C: 30 s, **51 °C**: 30 s, 72 °C: 1 min) × 35 cycles	*Aplosporella*, *Dothiorella*, *Phaeobotryon*
LSU	LR0R/LR5	(95 °C: 45 s, **55 °C**: 45 s, 72 °C: 1 min) × 35 cycles	*Phaeobotryon*
*tef1*-α	EF1-728F/EF1-986R	(95 °C: 15 s, **55 °C**: 20 s, 72 °C: 1 min) × 35 cycles	*Aplosporella*, *Dothiorella*, *Phaeobotryon*
*tub2*	Bt2a/Bt2b	(95 °C: 30 s, **55 °C**: 30 s, 72 °C: 1 min) × 35 cycles	*Dothiorella*
	T1/Bt2b		

### ﻿Phylogenetic analyses

The sequences obtained in this study were supplemented with additional sequences obtained from GenBank (Suppl. material 1) based on BLAST searches and from relevant published literature on the related genera (Bezerra et al. 2021; Wijayawardene et al. 2021; Xiao et al. 2021; Zhang et al. 2021; Peng et al. 2023). The individual data-sets of each gene region were aligned separately using MAFFT v. 6.0 (Katoh and Standley 2013) and trimmed at both terminal ends in MEGA v. 6.0 (Tamura et al. 2013). Maximum Likelihood (ML) analyses were conducted for the single gene sequence data sets (ITS and *tef1*-α regions for *Aplosporella*; ITS, *tef1*-α, and *tub2* regions for *Dothiorella*; ITS, LSU, and *tef1*-α regions for *Phaeobotryon*). Then the combined data set of each genus of all gene regions were used for multi-gene phylogenetic analyses including Maximum Likelihood (ML) and Bayesian Inference (BI) analyses. *Alanomycesindica* (CBS 134264), *Lasiodiplodiaamericana* (CFCC 50065), and *Alanphillipsiaaloeicola* (CBS 138896) were selected as the outgroup taxa for *Aplosporella*, *Dothiorella*, and *Phaeobotryon* analyses respectively.

Maximum Likelihood (ML) analyses were conducted using PhyML v. 3.0 (Guindon et al. 2010), employing a GTR model of site substitution with 1000 bootstrap replicates (Stamatakis 2014). Bayesian Inference (BI) analyses were conducted based on the DNA dataset from the results of the MrModeltest v. 2.4 (Nouri et al. 2004) using a Markov Chain Monte Carlo (MCMC) algorithm in MrBayes v. 3.1.2 (Ronquist and Huelsenbeck 2003). Two MCMC chains were run from random trees for 1,000,000 generations, resulting in a total of 10,000 trees. The first 25% of trees sampled were discarded as the burn-in phase of each analysis. The posterior probabilities (BPP) were calculated from the remaining trees (Rannala and Yang 1996). Phylogenetic trees were shown using FigTree v .1.4.4 (Rambaut 2018) and processed by Adobe Illustrator 2019.

## ﻿Results

### ﻿Phylogenetic analyses

The BLAST results indicated that the 13 isolates in this study resided in *Aplosporella*, *Dothiorella*, and *Phaeobotryon*. Datasets for the three genera, the number of characters of each gene with gaps and the substitution models used for BI analyses are provided in Table 2. The topologies of BI analyses did not significantly differ from the ML analyses.

**Table 2.** Substitution models used for Bayesian analyses in this study.

Analyses	Number of ingroup sequences	outgroup	Substitution models used for Bayesian analyses/Number of characters with gaps			
			ITS	LSU	tef1	tub2
*Aplosporella* 2-genes	24	*Alanomycesindica* CBS 134264	SYM+G /553	–	GTR+G /417	–
*Dothiorella* 3-genes	66	*Lasiodiplodiaamericana*CFCC 50065	GTR+I+G /494	–	GTR+G /322	GTR+I+G /448
*Phaeobotryon* 3-genes	36	*Alanphillipsiaaloeicola* CBS 138896	GTR+I /488	HKY+I /562	HKY+G/299	–

#### ﻿Species of *Aplosporella*

**Figure 1.**
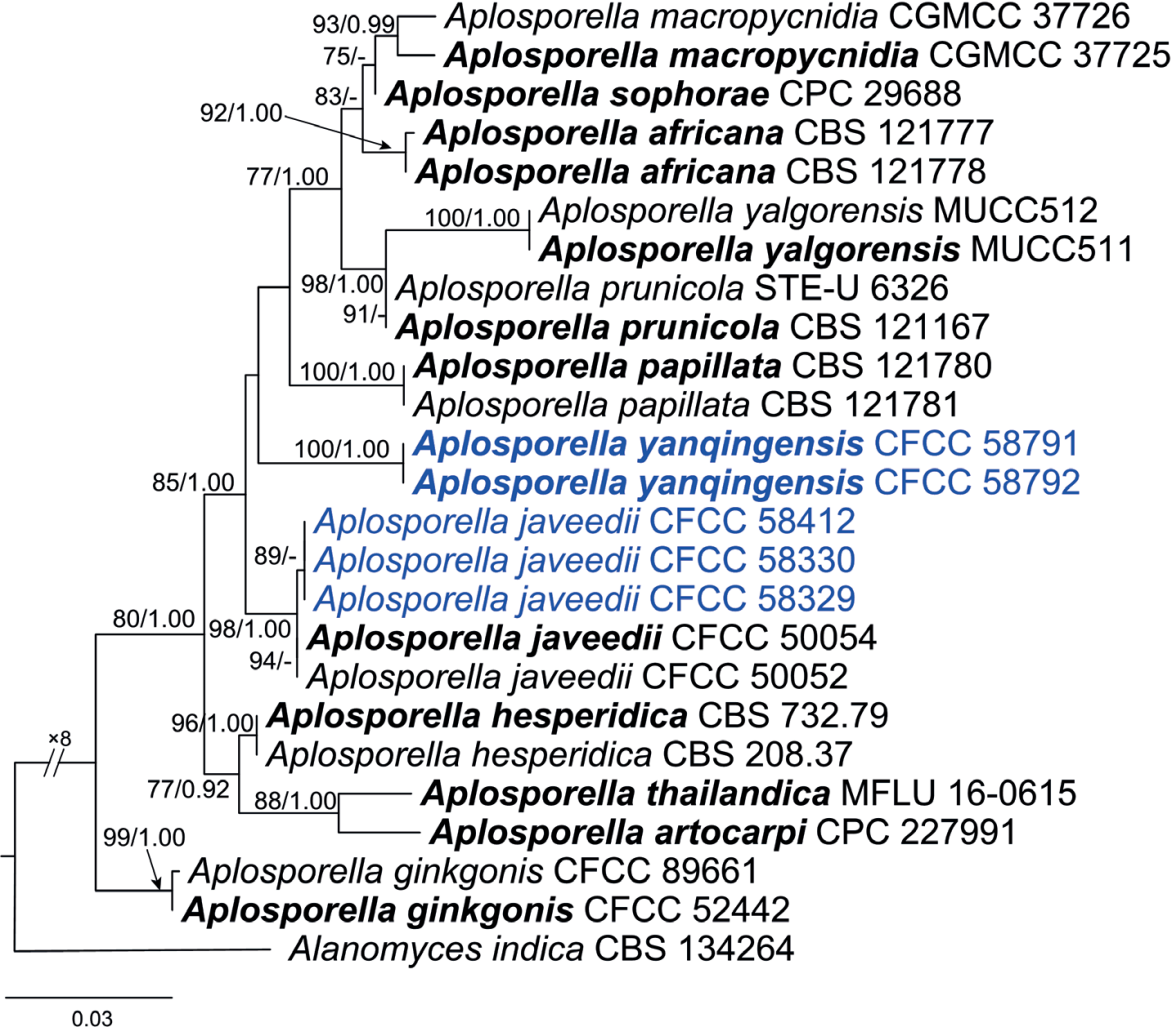
Phylogram of *Aplosporella* resulting from a maximum likelihood analysis based on combined ITS and *tef1* loci. Numbers above the branches indicateML bootstrap values (ML-BS ≥ 70%) and Bayesian Posterior Probabilities (BPP ≥ 0.9). The tree is rooted with *Alanomycesindica* CBS 134264. Ex-type isolates are in bold. Isolates from the present study are marked in blue.

Five isolates clustered into two phylogenetic groups for the individual genes (ITS and *tef1*-α), as well as the combined gene dataset (Fig. 1). In ML analysis based on the combined gene dataset, the matrix had 221 distinct alignment patterns. Estimated base frequencies are as follows: A = 0.211117, C = 0.277509, G = 0.253698, T = 0.257676; substitution rates: AC = 3.242352, AG = 4.568839, AT = 2.135067, CG = 2.137396, CT = 5.690231, GT = 1.000000; gamma distribution shape parameter: α = 0.217402. The isolates CFCC 58330, 58329, and 58412 resided in a clade with *Aplosporellajaveedii* (ML/BI = 98/1.00), while the isolates CFCC 58791 and 58792 formed an individual clade distinct from the other species in *Aplosporella* (ML/BI = 100/1.00).

#### ﻿Species of *Dothiorella*

**Figure 2.**
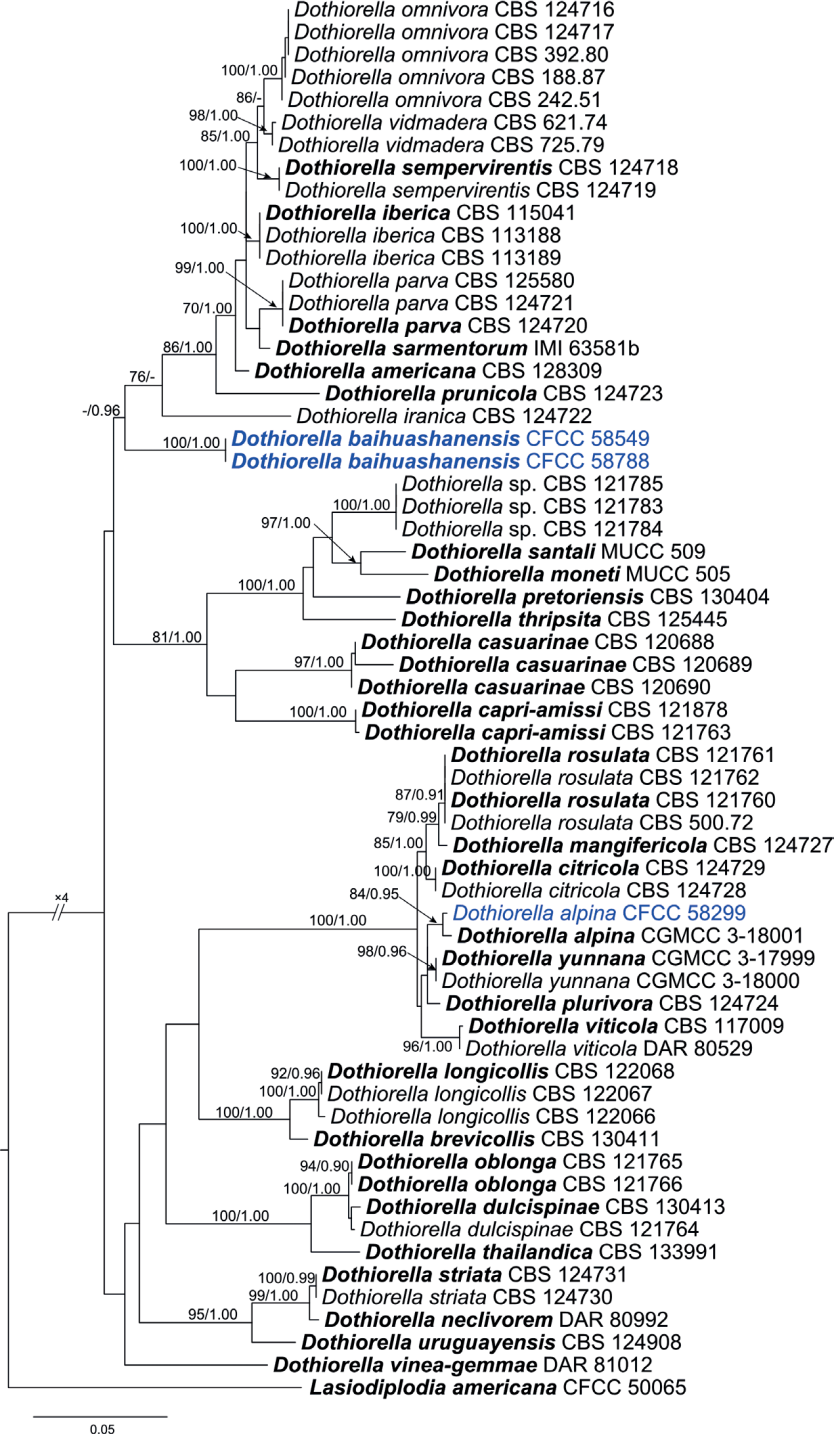
Phylogram of *Dothiorella* resulting from a maximum likelihood analysis based on combined ITS, *tef1* and *tub2* loci. Numbers above the branches indicateML bootstrap values (ML-BS ≥ 70%) and Bayesian Posterior Probabilities (BPP ≥ 0.9). The tree is rooted with *Lasiodiplodiaamericana*CFCC 50065. Ex-type isolates are in bold. Isolates from the present study are marked in blue.

Three isolates clustered in two clades for the individual genes (ITS, *tef1*-α, and *tub2*), as well as the combined gene dataset (Fig. 2). In ML analysis based on the combined gene dataset, the matrix had 478 distinct alignment patterns. Estimated base frequencies are as follows: A = 0.203201, C = 0.315247, G = 0.248158, T = 0.233395; substitution rates: AC = 0.994643, AG = 2.280369, AT = 1.123589, CG = 0.895887, CT = 4.309165, GT = 1.000000; gamma distribution shape parameter: α = 0.210467. The isolate CFCC 58299 grouped with *Do.alpina* (ML/BI = 84/0.95), while the isolates CFCC 58549 and 58788 formed a distinct clade from the other species (ML/BI = 100/1.00).

#### ﻿Species of *Phaeobotryon*

**Figure 3.**
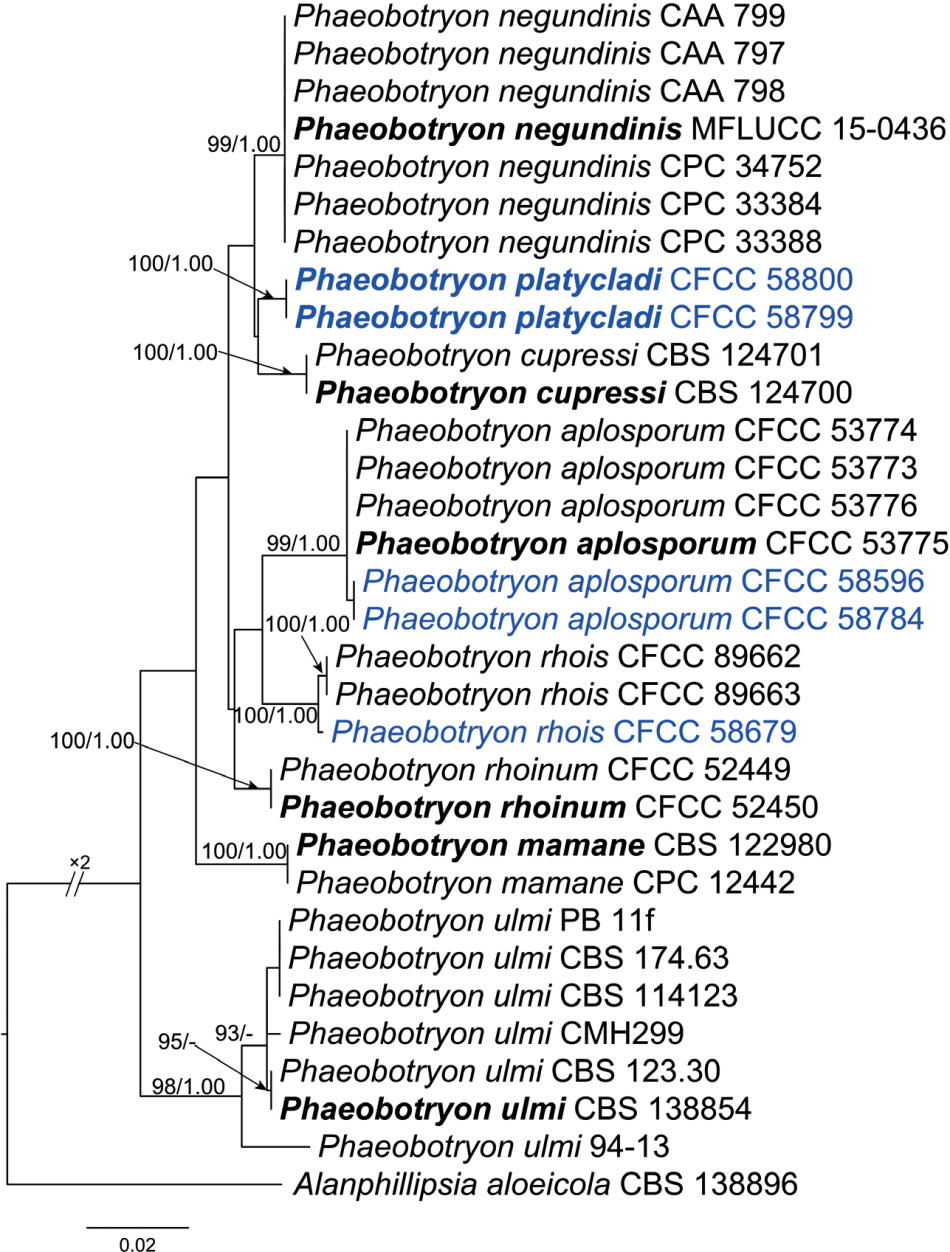
Phylogram of *Phaeobotryon* resulting from a maximum likelihood analysis based on combined ITS, LSU, and *tef1* loci. Numbers above the branches indicateML bootstrap values (ML-BS ≥ 70%) and Bayesian Posterior Probabilities (BPP ≥ 0.9). The tree is rooted with *Alanphillipsiaaloeicola* CBS 138896. Ex-type isolates are in bold. Isolates from the present study are marked in blue.

Five isolates clustered into three clades for the individual genes (ITS, LSU, and *tef1*-α), as well as the combined gene dataset (Fig. 3). In ML analysis based on the combined gene dataset, the matrix had 223 distinct alignment patterns. Estimated base frequencies are as follows: A = 0.223233, C = 0.267753, G = 0.277657, T = 0.231357; substitution rates: AC = 0.862696, AG = 2.117465, AT = 0.455729, CG = 1.132740, CT = 4.957268, GT = 1.000000; gamma distribution shape parameter: α = 0.272408. The isolate CFCC 58679 grouped with *Ph.rhois* (ML/BI = 100/1.00). The isolates CFCC 58596 and 58784 formed a unique lineage distinct from, but related to *Ph.aplosporum* as their closest relatives (ML/BI = 99/1.00). The isolates CFCC 58799 and 58800 formed a clade of their own separating them from other *Phaeobotryon* lineages (ML/BI = 100/1.00).

### ﻿Taxonomy

Based on DNA sequences and morphology, seven species belonging to three genera were identified. Of these, *Aplosporellajaveedii*, *Dothiorellaalpina*, *Phaeobotryonaplosporum*, and *Ph.rhois* are known species. The remaining three species are identified as new species (*Aplosporellayanqingensis*, *Dothiorellabaihuashanensis*, and *Phaeobotryonplatycladi*) and described below. Collect information and notes of all seven species were provided.

#### ﻿ Aplosporella javeedii

Taxon classificationFungiBotryosphaerialesAplosporellaceae

Jami, Gryzenh., Slippers & M.J. Wingf., Fungal Biol. 118(2): 174 (2013)

5167B2EC-0896-542F-9224-0E81106F8072

##### Description.

See Fan et al. 2015.

##### Materials examined.

China, Yunnan Province, Kunming City, Panlong District, Jinma County, Bailongsi Town, 25°3'44"N, 102°45'22"E, on dead branches of *Populuscanadensis*, 11 August 2022, Lu Lin & Ziqiang Wu (BJFC CF20230101, living culture CFCC 58330). Beijing City, Mentougou District, G109 National Highway, 40°3'2"N, 115°52'58"E, on dead branches of *Populusbeijingensis*, 25 August 2022, Lu Lin & Xinlei Fan (BJFC CF20230102, living culture CFCC 58329). Changping District, Liucun Town, Wangjiayuan Village, 40°10'23"N, 116°4'9"E, on dead branches of Populusalbavar.pyramidalis, 22 September 2022, Lu Lin & Xinlei Fan (BJFC CF20230103, living culture CFCC 58412).

##### Notes.

*Aplosporellajaveedii* was first discovered on *Celtisafricana* and *Searsialancea* in South Africa (Jami et al. 2014). Fan et al. (2015), Zhu et al. (2018), and Pan et al. (2019) expanded the host range of *Aplosporellajaveedii* to more than ten host families in China. This species has not been reported outside South Africa and China.

#### ﻿ Aplosporella yanqingensis

Taxon classificationFungiBotryosphaerialesAplosporellaceae

L. Lin & X.L. Fan sp. nov.

A6F858E9-4A40-58A8-8F87-EADBDCD9716E

 847680

Fig. 4

##### Etymology.

Named after the collection site of the type specimen, Yanqing District in Beijing City.

**Figure 4.**
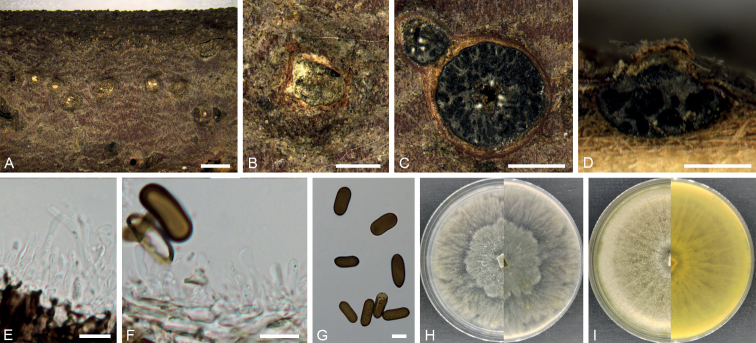
*Aplosporellayanqingensis* (BJFC CF20230104) **A, B** habit of conidiomata on twig **C** transverse section of conidiomata **D** longitudinal section through a conidioma **E, F** conidiogenous cells and paraphyses **G** conidia **H** colony on PDA at 14 days **I** colony on MEA at 14 days. Scale bars: 2 mm (**A**); 1 mm (**C**); 500 µm (**B, D**); 10 µm (**E–G**).

##### Description.

Conidiomata pycnidial, immersed to semi immersed, erumpent from bark surface, multilocular, 650–1500 μm in diam. Disc straw to greenish olivaceous, circular to ovoid, 350–650 μm in diam, with one central ostiole per disc. Ostioles inconspicuous, sometimes covered below disc by lighter entostroma, 100–300 µm in diam. Locules multiple, irregularly arranged, subdivided frequently by invaginations with common walls. Conidiophores reduced to conidiogenous cells. Conidiogenous cells hyaline, phialidic, 6.0–13.5 × 2.0–3.0 μm (av. ± S.D. = 10.7 ± 2.0 × 2.5 ± 0.2 μm). Paraphyses present, hyaline, smooth-walled, septate, unbranched, 26.5–37.5 × 2.0–3.0 μm (av. ± S.D. = 32.0 ± 3.5 × 2.4 ± 0.3 μm). Conidia aseptate, smooth, ellipsoid to subcylindrical, brown when mature, 16.0–21.5 × 6.0–9.5 μm (av. ± S.D. = 18.5 ± 1.3 × 7.7 ± 0.7 μm). Sexual morph not observed.

##### Culture characters.

Colonies on PDA spreading, white to pale grey, covering a 90 mm plate after 14 days at 25 °C. Colonies on MEA spreading, uniform with appressed aerial mycelium and crenate edge, upper white, reverse pale luteous covering a 90 mm plate after 14 days at 25 °C.

##### Materials examined.

China, Beijing City, Yanqing District, Yeyahu National Wetland Park, 40°24'55.43"N, 115°50'26.42"E, on branches of *Platycladusorientalis*, 25 July 2022, Yukun Bai & Xinlei Fan (holotype BJFC CF20230104, ex-holotype culture CFCC 58791); 40°24'55.46"N, 115°50'26.42"E, on branches of *Platycladusorientalis*, 25 July 2022, Yukun Bai & Xinlei Fan (paratype BJFC CF20230105, ex-paratype culture CFCC 58792).

##### Notes.

In the multi-gene analyses, *A.yanqingensis* is distinct and forms a moderately supported lineage clade (Fig. 1). In the ITS tree, *A.yanqingensis* shows a close relationship with a clade containing *A.africana* F.J.J. Van der Walt, Slippers & G.J. Marais, *A.macropycnidia* Dou & Y. Zhang ter, *A.papillata* F.J.J. Van der Walt, Slippers & G.J. Marais, *A.prunicola* Damm & Crous, *A.sophorae* Crous & Thangavel, and *A.yalgorensis* K.M. Taylor, P.A. Barber & T.I. Burgess. However, it differs from *A.africana* by longer conidia (18.5 × 7.7 vs. 14 × 8.5 μm) (Slippers et al. 2014), differs from *A.macropycnidia* by shorter paraphyses (32.0 × 2.4 vs. 38.4 × 2.9 μm) (Dou et al. 2017), differs from *A.papillata* by larger conidiogenous cells (10.7 × 2.5 vs. 7.4 × 2 μm) (Slippers et al. 2014), and differs from *A.prunicola* and *A.yalgorensis* by smaller conidia (18.5 × 7.7 vs. 20.2 × 11 for *A.prunicola* and 19.9 × 10.7 for *A.yalgorensis*) (Damm et al. 2007; Taylor et al. 2009). Besides, *A.yanqingensis* differs from *A.sophorae* by 25/528 in ITS region. Therefore, *A.yanqingensis* is introduced herein as a novel species. This is a new record of species in *Aplosporella* occurring on the host genus *Platycladus*.

#### ﻿ Dothiorella alpina

Taxon classificationFungiBotryosphaerialesBotryosphaeriaceae

(Y. Zhang ter. & Min Zhang) Phookamsak & Hyde, Asian Journal of Mycology 3(1): 168 (2020)

F0A301AE-2971-53B0-8FDF-3545D2D36F03

= Spencermartinsiaalpina Y. Zhang ter. & Ming Zhang, Mycosphere 7(7): 1058 (2016).

##### Description.

See Hyde et al. 2020.

##### Materials examined.

China, Yunnan Province, Diqing Tibetan Autonomous Prefecture, Shangri-La City, Sanba County, East Ring Road, 27°36'18"N, 100°1'19"E, on dead branches of *Populusszechuanica*, 9 August 2022, Lu Lin & Min Lin (BJFC CF20230106, living culture CFCC 58299).

##### Notes.

*Dothiorellaalpina* was first introduced by Zhang et al. (2016a) as *Spencermartinsiaalpina*, which has dark brown and 1-septate conidia. Hyde et al. (2020) transfer *S.alpina* to *Dothiorella* based on phylogenetic analyses of a concatenated dataset (ITS+*tef1*-α) and morphological similarity. *Dothiorellaalpina* was recorded on *Cirusunshiu* in Hunan Province, China, and *Platycladusorientalis* and *Ipomoea* sp. in Yunnan Province, China. In this study, a new record of *Do.alpina* from the host *Populusszechuanica* is included.

#### ﻿ Dothiorella baihuashanensis

Taxon classificationFungiBotryosphaerialesBotryosphaeriaceae

L. Lin & X.L. Fan sp. nov.

CB6DB2A7-D588-5F58-8699-D19771F30133

 847681

Fig. 5

##### Etymology.

Named after the collection site of the type specimen, Baihuashan Natural Scenic Area in Beijing City.

**Figure 5.**
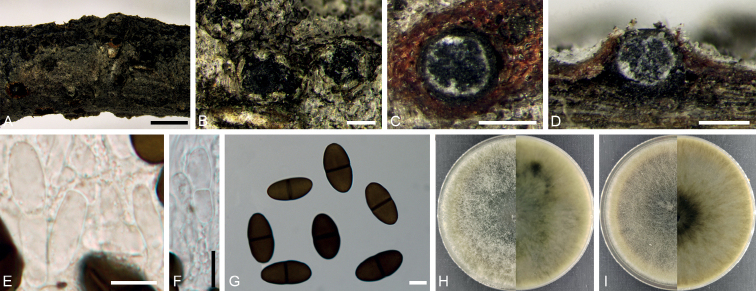
*Dothiorellabaihuashanensis* (BJFC CF20230107) **A, B** habit of conidiomata on twig **C** transverse section of a conidioma **D** longitudinal section through a conidioma **E, F** conidiogenous cells **G** conidia **H** colony on PDA at 14 days **I** colony on MEA at 14 days. Scale bars: 1 mm (**A**); 200 µm (**B–D**); 10 µm (**E–G**).

##### Description.

Conidiomata pycnidial, superficial or immersed, separate, ovoid, 350–500 µm in diam, occasionally aggregated into botryose clusters. Disc black, 200–300 µm in diam. Ostioles single, central, papillate. Conidiophores reduced to conidiogenous cells. Conidiogenous cells hyaline, holoblastic, cylindrical to subcylindrical or broadly lageniform, 7.5–16.0 × 3.5–6.5 μm (av. ± S.D. = 11.7 ± 2.2 × 4.6 ± 0.7 μm). Conidia1-septate, hazel to blackish brown, mostly truncate at the base and constricted at the septum or with a thickening at the base of the septum, moderately thick-walled, ovoid or oblong to ellipsoidal, 22.5–35.0 × 11.0–19.0 μm (av. ± S.D. = 27.9 ± 2.9 × 14.3 ± 2.2 μm).

##### Culture characters.

Colonies on PDA spreading, covering a 90 mm plate after 14 days at 25 °C, upper white to pale grey, reverse buff to dark grey. Colonies on MEA spreading, covering a 90 mm plate after 14 days at 25 °C, uniform with appressed aerial mycelium and crenate edge, upper white to pale grey, reverse honey to dark grey.

##### Materials examined.

China, Beijing City, Mentougou District, Qingshui County, Baihuashan Natural Scenic Area, 39°50'18.21"N, 115°34'21.13"E, on dead branches of *Juniperuschinensis*, 23 August 2022, Lu Lin & Xinlei Fan (holotype BJFC CF20230107, ex-holotype culture CFCC 58549); 39°50'18.16"N, 115°34'21.24"E, on dead branches of *Juniperuschinensis*, 23 August 2022, Lu Lin & Xinlei Fan (paratype BJFC CF20230108, ex-paratype culture CFCC 58788).

##### Notes.

The isolates CFCC 58549 and 58788 in this study formed a distinct linage in the phylogenetic trees of each individual gene (ITS, *tef1*-α, and *tub2*) and the combined gene dataset (Fig. 2). They were isolated from the branches *Juniperuschinensis*. *Dothiorellaiberica* was also recorded to host genus *Juniperus* (Alves et al. 2013). However, these two species are not closely related in our phylogenetic analysis.

#### ﻿ Phaeobotryon aplosporum

Taxon classificationFungiBotryosphaerialesBotryosphaeriaceae

M. Pan & X.L. Fan, Mycol. Prog. 18(11): 1356 (2019)

07EFD0C4-AB9F-566B-87DC-723A591C1825

##### Description.

See Pan et al. 2019.

##### Materials examined.

China, Beijing City, Mentougou District, Qingshui County, Baihuashan Natural Scenic Area, 39°51'11"N, 115°32'37"E, on dead branches of *Juglansmandshurica*, 23 August 2022, Lu Lin & Xinlei Fan (BJFC CF20230112, living culture CFCC 58596; BJFC CF20230113, living culture CFCC 58784).

##### Notes.

*Phaeobotryonaplosporum* was first discovered from *Rhustyphina* and *Syzygiumaromaticum* (Pan et al. 2019). It can be distinguished from other species in *Phaeobotryon* by its aseptate conidia (Pan et al. 2019). In this study, the conidia formed on the specimen BJFC CF20230112 are dark brick when mature, aseptate, (16.5–20.0 × 6.0–9.0 μm (av. ± S.D. = 18.3 ± 1.1 × 7.5 ± 0.8 μm), which overlap with the morphological characteristics described by Pan et al. (2019). Phylogenetically, the isolates CFCC 58596 and 58784 were clustered in a clade with *Ph.aplosporum* with high statistical support (ML/BI = 99/1). Therefore, the isolates CFCC 58596 and 58784 are identified as *Ph.aplosporum*. The current study extends its host range to *Juglansmandshurica*.

#### ﻿ Phaeobotryon platycladi

Taxon classificationFungiBotryosphaerialesBotryosphaeriaceae

L. Lin & X.L. Fan sp. nov.

18D6BBF3-3D15-5782-AF0F-79009C2235EF

 847682

Fig. 6

##### Etymology.

Named after the host genus, *Platycladus*.

**Figure 6.**
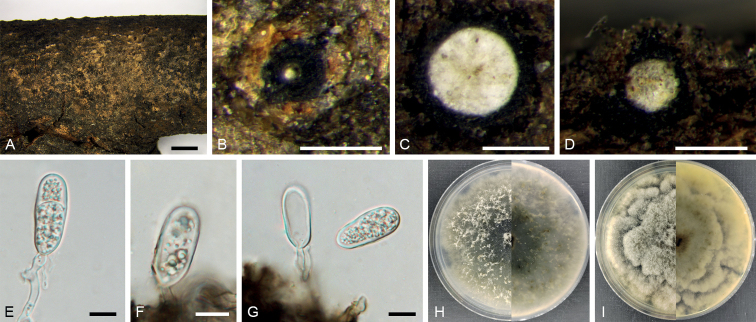
*Phaeobotryonplatycladi* (BJFC CF20230110) **A, B** habit of conidiomata on twig **C** transverse section of a conidioma **D** longitudinal section through a conidioma **E–G** conidiogenous cells and conidia **H** colony on PDA at 14 days **I** colony on MEA at 14 days. Scale bars: 2 mm (**A**); 200 µm (**B–D**); 10 µm (**E–G**).

##### Description.

Conidiomata pycnidial, scattered, subglobose to globose, erumpent, exuding faint yellow translucent conidial droplets from central ostioles, unilocular, 150–250 μm diam. Disc black, 80–200 µm in diam. Ostioles single, central, papillate, 21–35 µm. Conidiophores reduced to conidiogenous cells. Conidiogenous cells hyaline, smooth, thin-walled, cylindrical, holoblastic, phialidic, proliferating internally with visible periclinal thickening, 5.5–14.0 × 2.5–4.0 μm (av. ± S.D. = 10.2 ± 2.5 × 3.2 ± 0.4 μm). Conidia initially hyaline, oval, both ends broadly rounded, aseptate, rarely becoming 1-septate, 23.0–31.0 × 9.5–12.5 μm (av. ± S.D. = 26.2 ± 2.5 × 10.8 ± 0.8 μm).

##### Culture characters.

Colonies on PDA spreading, upper white to buff, reverse buff to isabelline covering a 90 mm plate after 14 days at 25 °C. Colonies on MEA spreading, stratiform, with appressed aerial mycelium and crenate edge, upper white to isabelline, reverse buff to hazel, covering a 90 mm plate after 14 days at 25 °C.

##### Materials examined.

China, Beijing City, Haidian District, National Botanic Gardens, 39°59'42.41"N, 116°12'47.24"E, on dead branches of *Platycladusorientalis*, 4 August 2022, Yukun Bai & Xinlei Fan (holotype BJFC CF20230110, ex-holotype culture CFCC 58799); 39°59'42.43"N, 116°12'47.46"E, on dead branches of *Platycladusorientalis*, 4 August 2022, Yukun Bai & Xinlei Fan (paratype BJFC CF20230111, ex-paratype culture CFCC 58800).

##### Notes.

*Phaeobotryonplatycladi* is monophyletic with *Ph.cupressi* in the phylogenetic tree without a significant statistical support. Conidial sizes of the two species overlap, but there are differences in 6/488 in ITS region, 3/556 in LSU region, and 18/293 in *tef1*-α gene with gaps.

#### ﻿ Phaeobotryon rhois

Taxon classificationFungiBotryosphaerialesBotryosphaeriaceae

C.M. Tian, X.L. Fan & K.D. Hyde, Phytotaxa 205(2): 95 (2015)

DEEC5BCA-A5EA-5B0F-8059-CB8819FEA59A

##### Description.

See Fan et al. 2015.

##### Materials examined.

China, Beijing City, Yanqing District, Zhangshanying County, 40°28'33"N, 115°49'58"E, on dead branches of Populusalbavar.pyramidalis, 16 September 2022, Lu Lin & Chengming Tian (BJFC CF20230109, living culture CFCC 58679).

##### Notes.

*Phaeobotryonrhois* was first discovered on *Rhustyphina* distributed in Ningxia Province, China (Fan et al. 2015). Pan et al. (2019) reported this species from *Dioscoreanipponica*, *Platycladusorientalis* and *Rhamnusdavurica* in Beijing, China. The current study extends its host range to Populusalbavar.pyramidalis.

## ﻿Discussion

In this study, a total of 13 isolates are identified as seven species of Botryosphaeriales, including three new species (*Aplosporellayanqingensis*, *Dothiorellabaihuashanensis*, and *Phaeobotryonplatycladi*) and four known species (*A.javeedii*, *Do.alpina*, *Ph.aplosporum*, and *Ph.rhois*). All three new species were isolated from coniferous trees: *A.yanqingensis* and *Ph.platycladi* from *Platycladusorientalis* and *Do.baihuashanensis* from *Juniperuschinensis*. Furthermore, the new records of *Do.alpina* from the host species *Populusszechuanica*, *Ph.aplosporum* from *Juglansmandshurica*, and *Ph.rhois* from Populusalbavar.pyramidalis are included.

The fungi of Botryosphaeriales play various ecological roles, such as saprotrophs, endophytes, or plant pathogens (Phillips et al. 2005, 2008, 2013; Luque et al. 2016). Some fungi exhibit strong pathogenicity, leading to severe diseases in different parts of various plants, such as *Botryosphaeriadothidea*, which can cause apple ring rot of stems and fruits (Zhang et al. 2016b), as well as poplar cankers (Li et al. 2019), and the dieback and leaf spot diseases of *Euonymusjaponicus* (Lin et al. 2023). Sometimes their ecological roles change, such as *Diplodiasapinea*, which is both an endophytic and a plant pathogenic fungus (Slippers et al. 2013). In this article, all species were isolated from diseased plant tissues, and their pathogenicity remains to be verified.

In this study, both *Dothiorella* and *Phaeobotryon* belong to Botryosphaeriaceae. Slippers et al. (2013) mentioned that some morphological features within Botryosphaeriaceae are not always stable, such as pigment production of conidia. These features might have already existed before the diversification of the group and have undergone further changes later (Slippers et al. 2013). In this study, only aseptate conidia were observed in *Phaerobotryonplatycladi*, and they may become pigmented with age. Moreover, whether septate or not seem to be an unstable characteristic throughout the genus *Phaerobotryon*. Phillips et al. (2013) mentioned that in most cases, the conidia of *Phaeobotryon* have two septa when mature. However, both the *Phaeobotryonaplosporum* observed in this study and the one described by Pan et al. (2019) have pigmented but without septa. *Phaeobotryonrhoinum* also shows pigmented and aseptate conidia (Daranagama et al. 2016). Other species of *Phaeobotryon* with pigmented and septate conidia are either saprobic or pathogenic, but *Ph.aplosporum* and *Ph.rhoinum* are both pathogenic (Rathnayaka et al. 2023). The phylogenetic state analysis of the trophic pattern, conidial colour, and separation of Botryosphaeriales conducted by Rathnayaka et al. (2023) indicate that this may correspond to nutritional mode.

## Supplementary Material

XML Treatment for Aplosporella javeedii

XML Treatment for Aplosporella yanqingensis

XML Treatment for Dothiorella alpina

XML Treatment for Dothiorella baihuashanensis

XML Treatment for Phaeobotryon aplosporum

XML Treatment for Phaeobotryon platycladi

XML Treatment for Phaeobotryon rhois

## Supplementary materials

Supplementary material 1 Strains used in the molecular analyses in this study Data type table (Excel spreadsheet) https://binary.pensoft.net/file/842805This dataset is made available under the Open Database License (http://opendatacommons.org/licenses/odbl/1.0/). The Open Database License (ODbL) is a license agreement intended to allow users to freely share, modify, and use this Dataset while maintaining this same freedom for others, provided that the original source and author(s) are credited.Author: Lu Lin
